# Social network characteristics and cervical cancer screening among Quechua women in Andean Peru

**DOI:** 10.1186/s12889-016-2878-3

**Published:** 2016-02-24

**Authors:** John S. Luque, Samuel Opoku, Daron G. Ferris, Wendy S. Guevara Condorhuaman

**Affiliations:** Department of Public Health Sciences and Hollings Cancer Center, Medical University of South Carolina, 135 Cannon Street, Ste. 303, MSC 835, Charleston, SC 29425 USA; Georgia Southern University, Jiann-Ping Hsu College of Public Health, Statesboro, GA USA; Augusta University, GRU Cancer Center, Augusta, GA USA; CerviCusco, Cusco, Peru

## Abstract

**Background:**

Peru has high cervical cancer incidence and mortality rates compared to other Andean countries. Therefore, partnerships between governmental and international organizations have targeted rural areas of Peru to receive cervical cancer screening via outreach campaigns. Previous studies have found a relationship between a person’s social networks and cancer screening behaviors. Screening outreach campaigns conducted by the nonprofit organization CerviCusco created an opportunity for a social network study to examine cervical cancer screening history and social network characteristics in a rural indigenous community that participated in these campaigns in 2012 and 2013. The aim of this study was to explore social network characteristics in this community related to receipt of cervical cancer screening following the campaigns.

**Methods:**

An egocentric social network questionnaire was used to collect cross-sectional network data on community participants. Each survey participant (ego) was asked to name six other women they knew (alters) and identify the nature of their relationship or tie (family, friend, neighbor, other), residential closeness (within 5 km), length of time known, frequency of communication, topics of conversation, and whether they lent money to the person, provided childcare or helped with transportation. In addition, each participant was asked to report the nature of the relationship between all alters identified (e.g., friend, family, or neighbor). Bivariate and multivariate analyses were used to explore the relationship between Pap test receipt at the CerviCusco outreach screening campaigns and social network characteristics.

**Results:**

Bivariate results found significant differences in percentage of alter composition for neighbors and family, and for mean number of years known, mean density, and mean degree centrality between women who had received a Pap test (*n* = 19) compared to those who had not (*n* = 50) (*p*’s < 0.05). The final logistic regression model was statistically significant (χ2 (2) = 20.911, *p* < .001). The model included the variables for percentage of family alter composition and mean density, and it explained 37.8 % (Nagelkerke *R*^2^) of the variance in Pap test receipt, correctly classifying 78.3 % of cases. Those women with higher percentages of family alter composition and higher mean density in their ego networks were less likely to have received a Pap test at the CerviCusco campaigns.

**Conclusions:**

According to this exploratory study, female neighbors more than family members may have provided an important source of social support for healthcare related decisions related to receipt of a Pap test. Future studies should collect longitudinal social network data on participants to measure the network effects of screening interventions in rural indigenous communities in Latin American countries experiencing the highest burden of cervical cancer.

## Background

Cervical cancer ranks third as the most common cause of cancer death affecting women in developing countries [1]. In South America the overall incidence rate is 20.3 per 100,000, but in Peru, the rate is 32.7 per 100,000 [2]. Likewise, Peru has higher cervical cancer death rates of 12.0 per 100,000 compared to other Andean countries in the region such as Colombia where the rate is 8.0 per 100,000 and Chile where the rate is even lower at 6.0 per 100,000 [2]. In terms of raw numbers, in Peru there are approximately 4,446 incident cervical cancer cases and 2,098 deaths annually, and cervical cancer causes more cancer deaths than any other cancer among women [3]. While in more developed countries, cervical cytology (Pap tests) and appropriate follow-up procedures are readily available and accessible, in resource poor countries such as Peru, mortality rates remain relatively high in comparison to other countries in the region. This situation is partly the result of inadequate technical and public health infrastructure to support high-quality Pap tests with reliable follow-up [1]. Cervical cancer screening in Peru reaches approximately 51 % of the eligible population [4].

Women who participate the least in cervical cancer screening in Peru are more likely to have limited formal education, low socioeconomic status, speak an indigenous language, live in a rural area, and be uninsured [5]. For many indigenous Quechua women in rural Andean Peru, access to healthcare facilities in larger cities and towns is limited because of transportation challenges. There is also the perception among Quechua inhabitants in the region that government healthcare facilities have inconvenient hours of operation and long wait times [6]. Other barriers to care include psychosocial and cultural beliefs related to embarrassment and fear of the Pap test [7]. Quechua women do however visit larger towns on weekend market days to sell their products from terrace farming and livestock or to purchase supplies. This presents an opportunity to engage the population with screening opportunities. One cost-effective method to address access barriers for rural populations is to provide mobile medical clinics to reach women with needed services [8]. A recent study to evaluate this outreach model in rural Peru found that women screened in mobile clinic tents in marketplaces reported that it was easier to obtain a Pap test than those who attended a stationary clinic in a building such as a health department facility [9].

There is some evidence to support the effect of social networks on cancer screening practices in the U.S.; however, a 2015 systematic review of studies on social networks and health conducted in low and middle income countries did not identify any studies examining cancer screening practices [10]. One U.S. study of mammography screening found that subjective norms and encouragement by family and friends to obtain a mammogram at baseline were associated with screening adherence at follow-up [11]. In another U.S. study on colorectal cancer screening, individuals who were most socially connected were more likely to have obtained colorectal cancer screening [12]. In one U.S. study with diverse Hispanic populations, Pap test and mammography utilization was positively associated with social integration [13], and in another study, Latina women’s daughters and female friends were important sources of influence for receiving a mammogram [14]. Finally, in a study of breast and cervical cancer screening practices among older, low-income Mexican-American women, the number of close friends was a predictor of both Pap test and mammography utilization [15].

The aim of this study was to explore social network characteristics related to cervical cancer screening receipt at screening campaigns in a rural indigenous community following CerviCusco Pap test screening outreach campaigns in 2012 and 2013. CerviCusco is a nonprofit clinic providing cervical cancer screening to approximately 10,000 women per year through both its clinic in Cusco and screening campaigns in surrounding towns and regions. It was hypothesized that the likelihood of a woman to have received a Pap test at the CerviCusco screening outreach campaigns would be related to her social network characteristics.

## Methods

### Setting

Quispicanchi is one of 13 provinces in the Cusco region in the Peruvian southern highlands. Over 70 % of the population speaks Quechua as their primary language [16]. The district of Quiquijana in Quispicanchi province has 10,340 residents and 86 % are classified as rural. The cervical cancer screening campaigns in 2012 and 2013 were held in the urban center of Quiquijana at the *Centro de Salud* (“Health Department”). However, other opportunities to receive Pap tests were available periodically at the same health department through government programs.

### Social network survey design

The purpose of carrying out the egocentric social network study was to identify “influencers” regarding health information in women’s social networks and to analyze social network characteristics which might be related to a woman’s history of receiving a Pap test [17]. In this ego network study, the “ego” was the survey participant and the “alters” were the actors with whom they reported a relationship or “tie.” We selected Quiquijana because there had been two previous campaigns where women had the opportunity to be screened there, and we wanted to focus our efforts on a Quechua speaking community where language barriers to health information exist.

One campaign was held in May 2012 and yielded 300 women, and then a subsequent campaign was held in June 2013 which yielded 100 women. We worked with CerviCusco staff to coordinate a social network survey conducted in Quechua by a trained interviewer from the community of Quiquijana. The interviewer’s family operated a local pharmacy in town, but she worked as a nurse in Cusco. We provided the interviewer with a list of 17 women who had attended both of the previous campaigns from two adjacent towns in the district of Quiquijana to generate the respondent-driven sample. The rationale for selecting women who had attended both campaigns was to identify women who might have more familiarity with cervical cancer screening and be more likely to have social networks of women up to date with screening as a result of the campaigns. Next, the interviewer was able to identify and survey 7 of these women, who as part of the survey each named six female friends, family members, or neighbors (alters). Three other women in the initial respondent driven sample had only attended one campaign. Each of the six women identified by the original sample of 10 women was then also contacted and surveyed by the interviewer for a total of 69 surveys (one survey was identified as a duplicate and not included in the subsequent analysis). Given the difficulty of locating alters using the respondent driven sampling technique, a sample size of 70 participants had been set a priori for the social network survey. When the participant could not be reached, the interviewer relied on her personal networks to recruit survey participants.

### Survey questions

A standard social network questionnaire was used to collect egocentric network data on community participants [18]. Each participant (ego) was asked to name six women they knew (alters) and identify the nature of their relationship or tie (family, friend, neighbor, other), residential closeness (within 5 km), length of time known, frequency of communication (daily, weekly), topics of conversation (family, politics, town gossip, health, work), and whether they lent money to the person, provided childcare or helped with transportation. In addition, each participant was asked to report the nature of their relationship or ties between all alters identified (e.g., friend, family, or neighbor). Participants also reported demographic information including age, years of formal education, marital status, average weekly income, family size, acculturation status based on a 4-item Spanish language acculturation scale [19], and wealth based on an 8-item Guttman household wealth scale [20].

### Data analysis

To analyze social network data, all survey data were entered into the EgoNet software program and exported to SPSS for further analysis [21]. Density was calculated based on the mean of the closeness variable between each alter pair identified by ego (0 = “no tie”; 1 = “tie”). Mean degree centrality was calculated as the average number of direct ties between alters. Betweenness centrality refers to ties between network members that are only possible through a third person or node [22]. Mean betweenness centrality was calculated as the average values of betweenness centrality between alters. Calculation of the percentage of alters in each ego network was based on named categories - family, friend, neighbor and other – of each alter. Descriptive statistics were generated for demographic characteristics. For continuous variables, bivariate associations using t-tests were used to detect differences in network characteristics between women who had received a Pap test at the CerviCusco campaigns compared to those who did not receive one. For categorical variables, Fisher’s Exact Test was used to detect statistically significant differences between groups. Statistical significance was set at an alpha level of .05 for two-sided tests. Social network variables with *p*-values less than 0.10 in the bivariate analysis were included in the logistic regression analysis. A logistic regression was performed to assess the effect of percentage of neighbor alter composition, percentage of friend alter composition, percentage of family alter composition, mean number of years known, mean degree centrality, and mean network density on the likelihood of receiving a Pap test at the CerviCusco campaign. All participants provided verbal informed consent and received a gift item (e.g., shawl) for completing the survey. The study was approved by the Institutional Review Boards of Georgia Southern University and Georgia Regents University and the Institutional Ethics Committee of Research within the National Institute of Health of the Peruvian Ministry of Health.

## Results

### Demographic and health characteristics

The majority of women surveyed lived in the town of Quiquijana (50, 72 %), and most of the other women lived in the nearby community of Accopata. The average age of participants was 37 years (SD = 7.63, range 21–59). The majority of women were either married (*n* = 27, 39 %) or living together with their male partner (*n* = 34, 49 %) (Table 1). Regarding work status, 32 (46 %) women were working and of this number, 21 (30 %) women worked full-time and 11 (16 %) worked part-time. Occupations were primarily in agriculture (*n* = 7, 10 %) and shops (*n* = 18, 26 %). There were also three teachers and one secretary. Husbands’ occupations were primarily in agriculture (*n* = 31, 44 %), and others worked as construction workers, drivers, teachers, and shop owners. On the 8-item Guttman wealth scale the average score was 5.58 (SD = .96), suggesting that residents had most of their essential needs met such as running water, indoor plumbing, and electricity. Education levels varied, with 13 (19 %) women having no formal education, 21 (30 %) having primary school only, 21 (30 %) completing secondary education, and 14 (20 %) having post-secondary education. The median income was between 201 and 400 soles per month (approximately $67 to $133 USD). The average household size was four people (SD = 1.37). Fifty-nine (86 %) participants spoke Quechua as their first language. The mean score on the Spanish language acculturation index was 1.03 (SD = .82) out of a possible score of 4 (completely Spanish language acculturated), indicating a preference for the Quechua language.

**Table 1:** Demographic Characteristics and Pap Test History of Survey Participants

Characteristic	Total (*N* = 69)
Age, years	37 (21–59)
Years of schooling	7.2 (0–16)
# People in household	4.4 (2–9)
Median income category/month (*soles*)	S/. 201–400
Score on Guttman wealth scale^a^ (0 = low to 8 = high)	5.6 (2–7)
Score on Spanish language acculturation scale^b^ (0 = low to 4 = high)	1.0 (0–3)
Marital status	
Married/living with a partner	61 (88 %)
Single/Widow	8 (12 %)
Currently employed	32 (46 %)
Residence	
Rent	14 (20 %)
Own	55 (80 %)
Pap test at CerviCusco campaign in 2012 or 2013	19 (28 %)
Self-reported Pap test	
1 year or less	30 (43 %)
> 1 yr. < 3 yrs.	17 (25 %)
> 3 yrs. < 5 yrs.	2 (3 %)
Never had a Pap test	20 (29 %)

*Notes*: Columns in mean values (ranges) for continuous variables and frequencies (percentages) for categorical variables

^a^ Modified version of a Guttman wealth scale with the following items: kitchen with gas, radio, TV, bicycle, refrigerator, plumbing, electricity, sanitation [20]

^b^ Modified version of the Acculturation Scale for Mexican Americans (ASMA) was used to measure acculturation (substituting “Quechua” for “English” language), with a value of “4” meaning completely Spanish language acculturated [19]

The majority of women (71.4 %) had received a Pap test in their lifetimes. Of these 50 women, 28 women had received a Pap test in 1 year or less, 17 between 1 and 3 years, 2 between 3 and 5 years, and 3 had only received one Pap test. The majority (*n* = 39, 57 %) had received their Pap tests in the Quiquijana *Centro de Salud*, whereas others received one in other health departments (2), private clinics (6), nonprofits clinics (2), or hospitals (2). After a review of CerviCusco records, 13 women in the sample had received a Pap test at the 2012 CerviCusco campaign, and 13 women received one at the 2013 campaign, for a total of 19 (28 %) women in the sample if both years were included, since seven women attended the campaigns both years. None of these women were diagnosed with an abnormal Pap test finding.

### Social network characteristics

For the social network characteristics, participants had a higher proportion of family members listed among the six alters named than the other relationship categories. There were 42 % of alters listed as family, 26 % as neighbors, and 15 % as friends. Participants listed health as a topic of discussion among one-third of alters (i.e., health was a discussion topic with two out of six alters). The mean number of years known between egos and alters was 20.8 years. The mean degree centrality value was 4.2, mean betweenness centrality was 0.2, and mean density was 0.8 (Table 2). The social network questions regarding social support resulted in very low frequency responses with lending money the highest (6–10 % of alters), followed by childcare (1–6 % of alters), and transportation (<1 % of alters).

**Table 2:** Ego Network Characteristics Based on 6 Alters

Characteristic	Total
Percent of alters - family	42
Percent of alters - friend	15
Percent of alters - neighbor	26
Percent of discussions - health	33
Number of years have known alters	20.8 (7.4)
Frequency of communication with alters^a^	1.6 (0.5)
Distance between alters^b^	0.6 (0.4)
Density^c^	0.8 (0.3)
Degree centrality	4.2 (1.3)
Betweenness centrality	0.2 (0.4)

*Notes*: Columns in mean values (SD) for continuous variables and percentages for categorical variables

^a^Frequency of communication with alters, 1 = “daily”; 2 = “1 time/week”; 3 = “2 times/week”; 4 = “3 times/week”

^b^Distance between alters, 0 = “less than 5 km”; 1 = “more than 5 km”

^c^Density calculated based on the mean of the closeness variable between each alter pair (0 = “no tie”; 1 = “tie”)

Results of independent samples t-tests found a significant difference between participants who had received a Pap test at the campaigns compared to participants who did not attend the campaigns based on (1) the percentage of alters who were identified as neighbors (20 % neighbor alter composition among those who did not receive a Pap test at the campaigns compared to 42.1 % neighbor alter composition among those who received one) (*p* = 0.01), and (2) as family members (51.3 % family alter composition among those who did not receive a Pap test compared to 16.7 % neighbor alter composition among those who did receive one) (*p* < .001). In addition, there were statistically significant differences on the variables of mean number of years known, mean density, and mean degree centrality between the two groups. There were no significant differences for alter composition percentages for those whom were identified as friends based on confirmed Pap test receipt. There were also no significant differences based on frequency of communication, physical distance, mean betweenness centrality, or percentage of alters sharing discussions on the topic of health (Table 3). For demographic characteristics the only significant difference was for mean number of years of schooling, with those who reported less education as more likely to have attended the CerviCusco campaign.

**Table 3:** Comparison of Demographic and Ego Network Characteristics by Confirmed Pap Test Receipt at CerviCusco Outreach Campaign

	Received Pap Test (*n* = 19)	Never Received Pap Test (*n* = 50)	*P* value
Demographic Characteristics			
Age	38.5	36.1	0.26
Number of people in household	4.8	4.3	0.19
Years of schooling	5.4	7.9	0.04*
Monthly income			
0–400 soles	13	27	0.41
401 soles and higher	6	23	
Marital status			
Married/living with a partner	19	42	0.06
Single/Widow	0	8	
Currently employed			
Yes	6	26	0.11
No	13	24	
Residence			
Own	14	40	0.40
Rent	5	10	
Guttman SES score	5.6	5.6	0.79
Spanish acculturation score	0.9	1.1	0.41
Ego Network Characteristics			
Percent of alters - family	16.7 %	51.3 %	0.00**
Percent of alters - friend	14.0 %	16.0 %	0.75
Percent of alters - neighbor	42.1 %	20.0 %	0.01**
Percent of discussions - health	26.8 %	35.7 %	0.23
Number of years have known alters	17.8	21.9	0.04*
Frequency of communication with alters	1.7	1.6	0.61
Distance between alters	0.7	0.6	0.19
Density	0.7	0.9	0.03*
Degree centrality	3.6	4.5	0.03*
Betweenness centrality	0.2	0.2	0.48

*Notes*: Columns in mean values for continuous variables and frequencies or percentages for categorical variables

*p < .05; **p < .01

Table 4 presents results of the final logistic regression model (χ2 (2) = 20.911, *p* < .001). The model explained 37.8 % (Nagelkerke *R*^2^) of the variance in Pap test receipt and correctly classified 78.3 % of cases with two variables included in the model. The results suggest that percentage of family alter composition and mean density are independently associated with the Pap test receipt. Specifically, women with higher percentages of family alter composition and higher mean density in their ego networks were less likely to have received a Pap test at the CerviCusco campaigns (OR = 0.025; 95 % CI = 0.002–0.251 and OR = 0.047; 95 % CI = 0.004–0.513).

**Table 4:** Logistic Regression Analysis of Confirmed Pap Test Receipt at CerviCusco Outreach Campaigns by Social Network Variables

Predictor	β	*SE* β	Wald’s χ^2^	*df*	*p*	OR	95 % CI
Constant	2.685	1.144	5.509	1	0.019	NA	
Percent of alters - family	−3.706	1.186	9.768	1	0.002	0.025	0.002–0.251
Density	−3.063	1.222	6.278	1	0.012	0.047	0.004–0.513
Test			χ^2^	*df*	*p*		
Goodness-of-fit test							
Hosmer & Lemeshow			12.932	7	0.074		

*Note*. Cox and Snell *R* ^2^ = .261. Nagelkerke *R* ^2^ = .378. NA = not applicable. Model χ^2^ (2) = 20.911, *p* < .001

## Discussion

This egocentric network study in a rural Quechua community in Andean Peru found that contrary to our study hypothesis, women who identified a higher proportion of neighbors in their immediate networks, not women whose networks had higher proportions of family or friends, were significantly more likely to have received a Pap test at one of the CerviCusco campaigns. The findings suggested that there was a significant difference in the percentage of family in one’s immediate network and ego network density for those who had received a Pap test at the CerviCusco campaigns compared to those who did not receive one. There were also differences in the percentage of neighbor members in one’s immediate network depending on screening status, but the variable was not included in the final logistic regression model. Those women who did not receive a Pap test reported higher percentages of family members in their immediate networks compared to women who had received one.

Previous studies have found that interpersonal influence on normative health behaviors – in this case screening – has a ripple effect and affects others to whom they are connected [23]. In this research study, data were not collected on alter’s cervical cancer screening history since this data would likely be unreliable. However, future studies should examine whether recently screened women might have a contagion effect on alters. Since the network question asked participants to name six women who were close to them, and participants had four options to name the alter’s appropriate category - either family, friend, neighbor, or other - there is the possibility that family-centric networks, although more common, did not serve as a protective effect or a positive influence when correlated with Pap test receipt at the CerviCusco campaigns. On the other hand, analysis of network data from the Framingham Heart Study data found that family networks were more important than friend or coworker networks for breast cancer screening - between sisters - and colorectal cancer screening - between spouses; however, no other effect was found for breast, prostate, or colorectal cancer screening [24].

Future research needs to examine the relationship between neighbors and family members in this community and the sources of support they provide. The most common type of support provided was monetary support, with 17 (25 %) participants identifying at least one alter who provided this type of support. However, only 10 (14 %) participants identified an alter who provided childcare support, and only 5 (7 %) participants identified an alter who assisted with transportation. These findings suggest that childcare and transportation needs were largely met or underreported, but occasionally alters could be relied on for lending money in times of need.

The significant finding for lower mean density of networks for women who received a Pap test at the CerviCusco campaigns compared to unscreened women with higher network density is difficult to interpret. The density measure differentiates those whose network members were more connected versus more diffuse networks. The result suggests a possible hypothesis that those women with denser networks might be less likely to attend a screening campaign than those whose networks were more diffuse or wide reaching. However, further research is necessary to interpret and understand this finding.

Because of the survey design, there were several study limitations. Because this was an egocentric network study and not a sociocentric network study, it was not possible to estimate ego’s network position in the whole network or structural characteristics of the whole network [18]. In addition, since the survey was cross-sectional, it was not possible to estimate how screening behaviors could influence alters over time. Finally, because of the small sample size, the study’s conclusions are limited since only six variables could be included in the logistic regression analysis based on the convention of one independent variable per 10 participants. Future studies with a larger sample size would permit a more robust statistical analysis of the findings.

## Conclusions

While there is the possibility that the finding for percentage of neighbors and family members in one’s egocentric network and cervical cancer screening status is a spurious finding due to the small sample size, if the finding is suggestive of a trend, then there are a number of possible explanations. Time constraints due to family commitments, such as childcare demands, have been cited in the literature as potential barriers to cervical cancer screening among Latinas [25, 26]. There is also the possibility that neighbors provided a more important source of social support for healthcare related decisions or for information about healthcare than friends or family. Future studies should collect longitudinal network data in rural indigenous communities in Latin America to measure the effect of screening interventions and better categorize these relationships to inform interventions such as peer education approaches. There is a need to disseminate and test evidence-based cervical cancer interventions in rural indigenous communities in Latin American countries experiencing the highest burden of cervical cancer, and social networks are one information channel that might be leveraged to deliver educational and behavioral interventions to increase adherence to screening and follow-up for abnormal findings.
